# On Multivalued Contractions in Cone Metric Spaces without Normality

**DOI:** 10.1155/2013/481601

**Published:** 2013-05-28

**Authors:** Muhammad Arshad, Jamshaid Ahmad

**Affiliations:** ^1^Department of Mathematics, International Islamic University, H-10, Islamabad 44000, Pakistan; ^2^Department of Mathematics, COMSATS Institute of Information Technology, Chak Shahzad, Islamabad 44000, Pakistan

## Abstract

Wardowski (2011) in this paper for a normal cone metric space (*X*, *d*) and for the family *𝒜* of subsets of *X* established a new cone metric *H* : *𝒜* × *𝒜* → *E* and obtained fixed point of set-valued contraction of Nadler type. Further, it is noticed in the work of Janković et al., 2011 that the fixed-point problem in the setting of cone metric spaces is appropriate only in the case when the underlying cone is nonnormal. In the present paper we improve Wardowski's result by proving the same without the assumption of normality on cones.

## 1. Introduction and Preliminaries

Huang and Zhang [1] generalized the notion of metric space by replacing the set of real numbers by ordered Banach space and defined cone metric space and extended Banach type fixed-point theorems for contractive type mappings. Subsequently, some other authors (e.g., see [2–15] and references therein) studied properties of cone metric spaces and fixed points results of mappings satisfying contractive type condition in cone metric spaces. Recently, Choa et al. [9], Kadelburg and Radenović [16], Klim and Wardowski [17], Latif and Shaddad [18], Radenović and Kadelburg [19], Rezapour and Haghi [20], and Wardowski [14, 21] obtained fixed points of set-valued mappings in normal cone metric spaces. On the other hand, it is shown in [11] that most of the fixed points results of mappings satisfying contractive type condition in cone metric spaces with a normal cone can be reduced to the corresponding results from metric space theory. The fixed-point problem in the setting of cone metric spaces is appropriate only in the case when the underlying cone is nonnormal, because the results concerning fixed points and other results in the case of cone metric spaces with nonnormal solid cones cannot be proved by reducing to metric spaces. In this paper, we prove the result of Wardowski [14] without the assumption of normality of cones. We need the following definitions and results, consistent with [1, 11, 14].

Let *E* be a Banach Space and *P* a subset of *E*. Then, *P* is called a cone whenever

- *P*  is closed, nonempty, and *P* ≠ {*θ*},
- *ax* + *by* ∈ *P* for all *x*, *y* ∈ *P* and nonnegative real numbers *a*, *b*,
- *P*∩(−*P*) = {*θ*}.

Each cone *P* induces a partial ordering ≼ on *E* by *x*≼*y* if and only if *y* − *x* ∈ *P*. So *x* < *y* will stand for *x*≼*y* and *x* ≠ *y*, while *x* ≪ *y* will stand for *y* − *x* ∈ int⁡*P*, where int⁡*P* denotes the interior of *P*. The cone *P* is called normal if there is a number *K* > 0 such that, for all *x*, *y* ∈ *E*,

(1) $$\begin{array}{c} \theta ≼x≼y\Rightarrow ||x||\leq K||y||. \end{array}$$

The least positive number *K*  satisfying (1) is called the *normal constant* of *P*.

**Definition 1**

Let *X* be a nonempty set. Suppose the mapping *d* : *X* × *X* → *E* satisfies

(d_1_) *θ*≼*d*(*x*, *y*) for all *x*, *y* ∈ *X* and *d*(*x*, *y*) = *θ* if and only if *x* = *y*,
(d_2_) *d*(*x*, *y*) = *d*(*y*, *x*) for all *x*, *y* ∈ *X*,
(d_3_) *d*(*x*, *y*)≼*d*(*x*, *z*) + *d*(*z*, *y*) for all *x*, *y*, *z* ∈ *X*.

Then, *d* is called a cone metric on *X*, and (*X*, *d*) is called a cone metric space.

Let (*X*, *d*) be a cone metric space, *x* ∈ *X* and {*x*_*n*_}_*n*≥1_ a sequence in *X*. Then, {*x*_*n*_}_*n*≥1_ converges to *x* whenever for every *c* ∈ *E* with *θ* ≪ *c* there is a natural number *N* such that *d*(*x*_*n*_, *x*) ≪ *c* for all *n* ≥ *N*. We denote this by lim⁡_*n*→*∞*_ *x*_*n*_ = *x* or *x*_*n*_ → *x*. {*x*_*n*_}_*n*≥1_ is a Cauchy sequence whenever for every *c* ∈ *E* with *θ* ≪ *c* there is a natural number *N* such that *d*(*x*_*n*_, *x*_*m*_) ≪ *c* for all *n*, *m* ≥ *N*.  (*X*, *d*) is called a complete cone metric space if every Cauchy sequence in *X* is convergent.

A set *A* ⊂ (*X*, *d*) is called closed if, for any sequence {*x*_*n*_}⊂*A*  convergent to *x*, we have *x*∈*A*. Denote by *N*(*X*) the collection of all nonempty subsets of *X* and by *C*(*X*) a collection of all nonempty closed subsets of *X*. Denote by Fix⁡*T* a set of all fixed points of a mapping *T*. In the present paper, we assume that *E* is a real Banach space, *P* is a cone in *E* with nonempty interior (such cones are called solid), and ≼ is a partial ordering with respect to *P*. In accordance with [14, Definition 3.1 and Lemma 3.1], we minutely modify the idea of  *H*-cone metric to make it more comparable with a standard metric.

**Definition 2**

Let (*X*, *d*) be a cone metric space and *𝒜*  be a collection of nonempty subsets of *X*. A map *H* : *𝒜* × *𝒜* → *E*  is called an *H*-cone metric on *𝒜* induced by *d* if the following conditions hold:

(H_1_) *θ*≼*H*(*A*, *B*)  for all *A*, *B* ∈ *𝒜*  and  *H*(*A*, *B*) = *θ*  if and only if *A* = *B*,
(H_2_) *H*(*A*, *B*) = *H*(*B*, *A*)  for all  *A*, *B* ∈ *𝒜*,
(H_3_) *H*(*A*, *B*)≼*H*(*A*, *C*) + *H*(*C*, *B*) for all  *A*, *B*, *C* ∈ *𝒜*,
(H_4_) If *A*, *B* ∈ *𝒜*, *θ*≺*ε* ∈ *E*  with  *H*(*A*, *B*)≺*ε*, then for each  *a* ∈ *A* there exists  *b* ∈ *B* such that  *d*(*a*, *b*)≺*ε*.

Examples can be seen in [14, examples 3.1 and 3.2].

## 2. Main Result

**Theorem 3**

Let (*X*, *d*)  be a complete cone metric space. Let *𝒜* be a nonempty collection of nonempty closed subsets of *X*, and let *H* : *𝒜* × *𝒜*→*E* be an *H*-cone metric induced by *d*. If for a map *T* : *X* → *𝒜*  there exists *λ* ∈ (0, 1) such that for all *x*, *y* ∈ *X*

(2) $$\begin{array}{c} H\left(Tx,Ty\right)≼\lambda d\left(x,y\right), \end{array}$$

then Fix⁡*T* ≠ *∅*.

**Proof**

Let *x*_0_ be an arbitrary but fixed element of *X* and *x*_1_ ∈ *Tx*_0_. If *x*_0_ = *x*_1_, then *x*_0_ ∈ Fix⁡*T*, and if *x*_0_ ≠ *x*_1_, using the fact that

(3) $$\begin{array}{c} H\left(Tx_{0},Tx_{1}\right)≼\lambda d\left(x_{0},x_{1}\right)≺\sqrt{\lambda }d\left(x_{0},x_{1}\right), \end{array}$$

we may choose *x*_2_ ∈ *X* such that *x*_2_ ∈ *Tx*_1_  and

(4) $$\begin{array}{c} d\left(x_{1},x_{2}\right)≺\sqrt{\lambda }d\left(x_{0},x_{1}\right). \end{array}$$

Similarly, in case *x*_1_ ≠ *x*_2_, we may choose *x*_3_ ∈ *X* such that *x*_3_ ∈ *Tx*_2_  and

(5) $$\begin{array}{c} d\left(x_{2},x_{3}\right)≺\sqrt{\lambda }d\left(x_{1},x_{2}\right)≺{\left(\sqrt{\lambda }\right)}^{2}d\left(x_{0},x_{1}\right). \end{array}$$

We can continue this process to find a sequence {*x*_*n*_} of points of *X*  such that

(6) xn+1∈Txn, n=0,1,2,…,d(xn,xn+1)≺λd(xn−1,xn)≺(λ)2d(xn−2,xn−1)≺⋯≺(λ)nd(x0,x1).

Now for any *m* > *n*,

(7) d(xm,xn)≼d(xn,xn+1)+d(xn+1,xn+2)+⋯+d(xm−1,xm)≼[λn/2+λ(n+1)/2+⋯+λ(m−1)/2]d(x0,x1)≼[λn/21−λ1/2]d(x0,x1).

Let *θ* ≪ *c*  be given. Choose a symmetric neighborhood *V* of *θ* such that *c* + *V*⊆int⁡*P*. Also, choose a natural number *N*_1_ such that [*λ* ^*n*/2^/(1 − *λ* ^1/2^)]*d*(*x*_0_, *x*_1_) ∈ *V*, for all *n* ≥ *N*_1_. Then, (*λ* ^*n*/2^/(1 − *λ* ^1/2^))*d*(*x*_1_, *x*_0_) ≪ *c*, for all *n* ≥ *N*_1_. Thus,

(8) $$\begin{array}{c} d\left(x_{m},x_{n}\right)≼\left[\frac{\lambda^{n/2}}{1-\lambda^{1/2}}\right]d\left(x_{0},x_{1}\right)\ll c, \end{array}$$

for all *m* > *n*. Therefore, {*x*_*n*_}_*n*≥1_ is a Cauchy sequence. Since *X* is complete, there exists *u* ∈ *X* such that *x*_*n*_ → *u*. Since

(9) $$\begin{array}{c} H\left(Tx_{n},Tu\right)≼\lambda d\left(x_{n},u\right)≺\sqrt{\lambda }d\left(x_{n},u\right), \end{array}$$

for each *n*, *x*_*n*+1_ ∈ *Tx*_*n*_, we have *y*_*n*_ ∈ *Tu* such that $d(x_{n+1},y_{n})≺\sqrt{\lambda }d(x_{n},u).\;$Now, choose a natural number *N*_2_  such that

(10) $$\begin{array}{c} d\left(x_{n},u\right)\ll \frac{c}{2}\;\forall n\geq N_{2}. \end{array}$$

Then for all *n* ≥ *N*_2_,

(11) d(u,yn)≼d(u,xn+1)+d(xn+1,yn)≼d(u,xn+1)+λd(xn,u)≼d(u,xn+1)+d(xn,u)≪c2+c2=c.

It follows that *y*_*n*_ → *u*, and it implies that *u* ∈ *Tu*.

**Example 4**

Suppose *X* = [0,1], *E* = *C*_*R*_^2^[0,1]  with the norm ||*x*|| = ||*x*||_*∞*_ + ||*x* ^/^||_*∞*_, *P* = {*x* ∈ *E* : *x* ≥ 0}, *x*(*t*) = *t*, and *y*(*t*) = *t* ^2*K*^. Then,  0 ≤ *x* ≤ *y*,  ||*x*|| = 2, and ||*y*|| = 1 + 2*K*. For all *K* ≥ 1, since *K*||*x*|| < ||*y*||. Therefore, *P* is non-normal. Define *d* : *X* × *X* → *E* as follows:

(12) $$\begin{array}{c} \left(d\left(x,y\right)\right)\left(t\right)=\left|x-y\right|e^{t}. \end{array}$$

Let *𝒜* be a family of subsets of *X* of the form *𝒜* = {[0, *x*] : *x* ∈ *X*} ∪ {{*x*} : *x* ∈ *X*}, and define *H* : *𝒜* × *𝒜* → *E* as follows:

(13) H(A,B)={|x−y|etfor  A=[0,x],  B=[0,y],  |x−y|etfor  A={x},  B={y},Max⁡{y,|x−y|}etfor  A=[0,x],  B={y},Max⁡{x,|x−y|}etfor  A={x},  B=[0,y].

It is easy to observe that *H*  satisfies (H_1_)–(H_4_) of Definition 2. Define *T* : *X* → *𝒜*  as

(14) $$\begin{array}{c} Tx=\{\begin{array}{cc} \left\{0\right\}, & \text{for}\;x\in \left[0,\frac{1}{2}\right],\\ \left[0,\frac{1}{2}{\left(x-\frac{1}{2}\right)}^{2}\right], & \text{for}\;x\in (\frac{1}{2},1]. \end{array} \end{array}$$

Note that *T* satisfies the conditions of Theorem 3 with *λ* = 1/2 and 0∈Fix⁡*T*.
